# Secondary Traumatization, Psychological Stress, and Resilience in Psychosocial Emergency Care Personnel

**DOI:** 10.3390/ijerph16173213

**Published:** 2019-09-03

**Authors:** Anja Greinacher, Alexander Nikendei, Renate Kottke, Jürgen Wiesbeck, Wolfgang Herzog, Christoph Nikendei

**Affiliations:** 1 Department of General Internal Medicine and Psychosomatics, University Hospital Heidelberg, 69115 Heidelberg, Germany; 2 German Red Cross, Rescue Service Bodensee-Oberschwaben, 88520 Ravensburg, Germany; 3 German Red Cross, Baden-Wuerttemberg Regional Association, 70372 Stuttgart, Germany

**Keywords:** psychosocial emergency care, secondary traumatization, psychological stress, resilience factors

## Abstract

Volunteers active in psychosocial emergency care offer psychological first aid to survivors of accidents and trauma, their relatives, eye witnesses, bystanders, and other first responders. So far, there are no studies that investigate the secondary and primary traumatization of this group of first responders. We included *N* = 75 volunteers, who filled out questionnaires to assess their secondary (QST/FST) and primary traumatization (PDS), and levels of comorbid psychological stress (PHQ-9, GAD-7, SF-12). We investigated factors of resilience by measuring attachment behavior (ECR-RD, RQ-2), level of personality functioning (OPD-SFK), sense of coherence (SOC-29), social support (F-SozU), and mindfulness (MAAS). The volunteers’ levels of secondary and primary traumatization were below cut-off scores. Their levels of comorbid psychological stress were comparable to representative norm samples. Additionally, the volunteers presented high levels of resilience. Gender (β = 0.26; *p* < 0.05), case discussions (β = −0.37; *p* < 0.05), and social support (β = 0.45; *p* < 0.01) were revealed to be predictors of secondary traumatization, while mindfulness turned out to be a predictor of primary traumatization (β = −0.34; *p* = 0.008). However, we cannot rule out that the low prevalence of traumatization and comorbid psychological stress in our study sample might not be explained by a positive response bias.

## 1. Introduction

First responders include policemen, firefighters, search and rescue personnel, emergency and paramedical teams. Usually, they are among the first people to arrive at a potential trauma site. Therefore, first responders are constantly exposed to emotionally challenging and unpredictable situations [1]. These situations may overwhelm the individuals’ capability to adapt and cope, leading to high stress levels and potential mental and physical changes [2]. The most frequently researched condition in first responders is post-traumatic stress disorder (PTSD). Apart from primary traumatization, first responders are also prone to secondary traumatization [3].

Volunteers in psychosocial emergency care represent a specific group of first responders enfolding all social classes. They give psychological support to accident or trauma survivors, their relatives, eye witnesses, first-aiders, passive bystanders, and other first responders after emergency responses [4]. Typical emergency responses of volunteers in psychosocial emergency care are, for example, giving support to injured people, helping people who received the news of a relative’s death, accompanying the police in the delivery of a death notice and subsequent care, and offering assistance after crib death. Their intervention immediately after potentially traumatic incidents serve to prevent immediate or chronic maladaptive psychosocial stress reactions [5]. During their emergency responses, the volunteers usually do not experience life-threatening situations, nor do they witness others in life-threatening situations; both are the main reasons for primary traumatization. However, even listening to the victims and offering support can lead to high levels of stress in volunteers in psychosocial emergency care. Therefore, this group may be at high risk of developing a condition termed secondary traumatization [6].

Secondary traumatization is caused by repeated or extreme confrontation with details of traumatic situations without any direct sensory impressions [7] and is described as the victims’ symptoms being transferred to another individual [8,9]. In the fifth edition of the Diagnostic and Statistical Manual of Mental Disorders (DSM-V), the etiological factor (experience of a traumatic event) was extended to include indirect exposure to a traumatic event. While some authors are against this addition due to the decreased discriminatory power of the diagnosis [10], other authors advocate the change as it depicts secondary traumatization [11]. Horesh [12] investigated whether the new diagnostic criteria actually correspond to clinical and research findings on secondary traumatization: Secondary traumatization depends on many variables other than just the knowledge of a traumatic event, such as empathy [13], emotional contagion [14], and biological factors [15]. Furthermore, observations of Holocaust survivors and their descendants have shown that traumatization can be passed on without ever talking about the traumatic events [16]. In the context of first responders, the symptoms of secondary traumatization are seen identically to those resulting from primary traumatization: Intrusive thoughts and memories, avoidance of people and places that might trigger memories of the event, and arousal [17]. Furthermore, there can be symptoms of depression and anxiety disorders [18], emotional exhaustion, depersonalization, and obsessive compulsive disorders. Additionally, harmful changes in professionals’ views of themselves, others, and the world, commonly referred to as vicarious traumatization, can occur [19].

Several studies have found secondary traumatization in first responders [3]. Hence, we assumed this also to be the case for volunteers in psychosocial emergency care services. However, so far, little is known about secondary traumatization and psychological stress in volunteers working in psychosocial emergency care.

Resilience is defined as “an outcome of successful adaptation to adversity” [20]. It is a separate, interdependent, dynamic process that is orthogonal to the presence or absence of symptoms and, therefore, does not merely describe the absence of a mental disorder or its symptoms [21]. It is a complex interaction between biological, psychological, social, and cultural factors. Which factors promote resilience differs depending on situation and context [22]. The resilience-based research approach focuses on “asking more complex questions regarding wellbeing, such as when, how, why, and for whom do resources truly matter” [23]. One of the best researched protective factors in the context of mental health in first responders is social support. A meta-analysis on first responders from Prati and Pietrantoni [24] showed a significant positive influence of social support on mental health, especially if the people affected perceived it as supportive. Factors of resilience that have been investigated in more detail are internal control, self-efficacy, mindfulness, and engagement [3]. However, there are no studies about factors of resilience in psychosocial emergency care.

Even though psychosocial emergency care has been professionalized in the past years, only little is known about secondary traumatization of volunteers working in this area. This study is the first to investigate the prevalence of secondary and primary traumatization in volunteers working in psychosocial emergency care. Furthermore, we were interested in comorbid psychological stress, as well as factors of resilience. We hypothesized that (I) volunteers in psychosocial emergency care would show similar symptoms to other groups of professionals working with traumatized individuals: Increased symptoms of secondary and primary traumatization, as well as (II) comorbid psychological stress (e.g., symptoms of depression and anxiety disorders). (III) We assumed that lower levels of psychological stress would be correlated with high levels of resilience, and that (IV) factors of resilience that correlate strongly with trauma symptoms would also be predictors of trauma symptoms.

## 2. Methods

This study was approved by the ethics committee of the University Hospital Heidelberg (ethics application no. S-139/2016) and all volunteers gave their informed consent to participate. Volunteers were able to withdraw their participation without any disadvantage. The study was conducted in accordance with the Declaration of Helsinki (most recent version: Fortaleza, Brazil, 2013).

In Germany in the early 1990s, several initiatives were developed which focused on the psychological support in situations of crisis or emergency of physically uninjured persons. These initiatives worked closely with the emergency services. In the following years, particularly the major help organizations and church organizations began to engage in psychosocial emergency care. Standards and qualifications of training within these organizations varied greatly. At the same time, the police and the fire services were increasingly integrated as initiators of psychosocial emergency care. Over time, the range of services offered to those affected was extended to include emergency personnel. Additionally, specialized training structures were developed with a support system for emergency personnel [4]. Psychosocial emergency care is a firmly established component of emergency strategies. In emergency situations (e.g., accidents, crib death, suicide), psychosocial emergency care is automatically contacted by the emergency call-takers and dispatchers. The volunteers have schedules and can be contacted around the clock during their working hours. The type and number of emergency responses can vary greatly among volunteers. All operations are carried out in groups of two. Following assignment, the volunteers first exchange information, attend a debriefing session with the supervisor, and finally draw up an assignment report. The volunteers receive training before they become active in psychosocial emergency care. Among other things, the participants receive information on the work in psychosocial emergency care (e.g., emergency response organization and logistics, legal bases), basic psychological and psychiatric knowledge, as well as communication training for crisis situations. In addition, aspects such as ‘dealing with death’ and self-care are covered during training. Case discussions and practical training, e.g., role play, are key training components. The training comprises three weekends over a period of six months. Between the second and third weekend, the volunteers start co-working in psychosocial emergency responses.

Study design and Procedure: We surveyed all volunteers currently working in the psychosocial emergency care of the German Red Cross in the regional association of the federal state of Baden-Württemberg in a cross-sectional design. After consulting the management of the psychosocial emergency care, the volunteers were recruited in the context of regular staff meetings in 5 of 24 district associations (Heidelberg, Balingen, Pforzheim, Ehingen, Ravensburg). Between June and December 2016, a research assistant visited all five district associations introducing the study and its aims to the volunteers. After giving their informed consent, all volunteers who were present at the meetings of their district association were invited to complete the questionnaires. In total, 111 volunteers working for the five different district associations were asked to participate in the study. During the visits, 79 volunteers (71.2%) attended the meetings, and all of them completed the questionnaires.

Seventy-nine volunteers working in psychosocial emergency care filled in the questionnaires. Table 1 displays the sociodemographic data of the study participants: 36.7% of the participants belonged to the district association of Heidelberg, 21.5% to Balingen, 20.3% to Pforzheim, 12.7% to Ehingen, and 8.9% to Ravensburg. Four cases were excluded due to extreme values in the outcome variables. These were determined using a box plot [25]. Finally, *N* = 75 volunteers were included in the analysis.

The following questionnaires were used:

Sociodemographic data. The following sociodemographic data were collected: Age, gender, nationality, marital status, graduation, and profession.

Data relating to work in psychosocial emergency care. Participants were asked to indicate how many years they had been engaged in psychosocial emergency care so far and in how many emergency responses they had participated. They were asked to specify the three emergency responses they had perceived as the most difficult, if they had received different forms of supervision and in which frequency, and how well prepared they felt for their emergency responses as regards knowledge, skills, and attitudes towards psychosocial emergency care.

Questionnaire for Secondary Traumatization (QST). Symptoms of a secondary traumatization were assessed using the German version of the Questionnaire for Secondary Traumatization (FST; [26,27]). This questionnaire comprises 31 questions which investigate symptoms of intrusion, avoidance, hyperarousal, parapsychotic sense of threat, and PTSD comorbidities, such as symptoms of depression. The questionnaire can yield scores from 31 to 155. Individuals with sum scores above 65 are seen as suffering from clinically relevant symptoms; scores between 65 and 82 indicate moderate secondary traumatization, and scores above 82 are categorized as severe secondary traumatization. We adapted the instructions for the questionnaire to the context of working as a volunteer in psychosocial emergency care, as well as the questions.

Post-traumatic Stress Diagnostic Scale (PDS). Primary traumatization was assessed by the German version [28] of the Post-traumatic Stress Diagnostic Scale (PDS; [29]). This questionnaire measures post-traumatic stress according to the Diagnostic and Statistical Manual of Mental Disorders (DSM-IV) and yields both a dichotomous result (PTSD+/PTSD−) and a symptom severity score. Each item can be answered on a Likert scale ranging from 0 to 3; the symptom severity score ranges from 0 to 51.

Patient Health Questionnaire (PHQ-9). Depression was assessed with the German version [30] of the depression module of the Patient Health Questionnaire (PHQ-9; [31]). The severity of depressive symptoms is determined by calculating the sum score, ranging between 0 and 27. Scores between 1 and 4 suggest minimal depressive symptoms, scores between 5 and 9 suggest mild depression, scores between 10 and 14 suggest moderate depression, and scores of 15 or above suggest severe depression. In a representative German norm sample, the mean sum score for depression was M = 3.60(SD = 4.08; [32]).

Generalized Anxiety Disorder Scale (GAD-7). The German version [33] of the Patient Health Questionnaire anxiety module, the Generalized Anxiety Disorder Scale (GAD-7; [34]), was used to assess symptoms of a generalized anxiety disorder. The sum score ranges from 0 to 21: Scores between 1 and 4 suggest minimal symptoms of an anxiety disorder, scores between 5 and 9 suggest a mild anxiety disorder, scores between 10 and 14 suggest a moderate anxiety disorder, and scores of 15 or above suggest a severe anxiety disorder. In a study with a representative population sample, the mean sum score of the GAD-7 was M = 2.95 (SD = 3.41; [33]).

Short Form Survey (SF-12). The Short Form Survey (SF-12, short version of the SF-36; [35]) measures health-related quality of life, which describes functionality and well-being from the patient’s point of view. The SF-12 shows good reliability and validity and consists of two of the eight SF-36 scales, namely the scales for physical (PCS) and mental quality of life (MCS). Sum scores range from 0 to 100 with M = 50.00, SD = 10.00.

Experiences in Close Relationships—Revised (ECR-RD). The German version (ECR-RD; [36]) of the questionnaire Experiences in Close Relationships—Revised [37] measures fear of attachment and avoidance of attachment in intimate relationships via 36 items. The ECR-RD shows (very) good reliability and validity. In a German non-clinical sample, the mean score for fear of attachment was M = 2.77 (SD = 1.09) and the mean score for avoidance of attachment was M = 2.36 (SD = 1.00).

Relationship Questionnaire (RQ-2; [38]). The Relationship Questionnaire assesses attachment to the self and others. The model of the self (positive/negative) and the model of the others (positive/negative) combined result in the attachment style. Standard values are not available.

OPD Structure Questionnaire short scale (OPD-SQS). The OPD Structure Questionnaire short scale (OPD-SQS; [39]) assesses personality dysfunction in accordance with the Operationalized Psychodynamic Diagnosis system (OPD-2). The questionnaire shows moderate correlations with clinical evaluations of experts regarding levels of personality functioning and is therefore valid in this area. The present study used the OPD-SQS 12-item short form (OPD-SQS) which has good psychometric characteristics [40]. Each item is rated on a 5-point scale, with higher scores indicating lower level of personality functioning. The sum score ranges from 0 (“highest level of personality functioning”) to 48 (“lowest level of personality functioning”). Subjects without psychotherapeutic treatment showed a mean sum score of M = 15.41, SD = 7.71.

Sense of Coherence Scale (SOC-29). The German version [41] of the Sense of Coherence Scale (SOC-29 [42]) measures the sense of coherence in individuals. After reversal of inverted items, the sum value is calculated, ranging from 29 to 203. In the most recent German norm sample, the mean score was M = 155.00 (SD = 23.00; [43]).

Questionnaire for Social Support Short Form (F-SozU K-14). The support that individuals perceived and anticipated to receive from their support from one’s environment was measured with the 14-item version of the Social Support Questionnaire (F-SozU K-14). The sum score ranges from 0 to 5. In a German norm sample, the mean score on the F-SozU K-14 was M = 3.9 (SD = 0.7; [44]).

Mindful Attention and Awareness Scale (MAAS). The Mindful Attention and Awareness Scale (MAAS; [45]) is an established, unidimensional questionnaire assessing the subjective experience of mindfulness in every-day life. The MAAS uses 15 items rated on a scale from 1 (“almost always”) to 6 (“almost never”). Scoring involves calculating mean performance across the 15 items, with higher scores indicating greater mindfulness. The German version was validated by Michalak et al. [46] and shows a good internal consistency (Cronbach’s α = 0.83).

Data Analysis: Four cases were excluded due to outliers in the data. Thus, *N* = 75 cases were evaluated in total. All data were coded and analyzed using SPSS Statistics 24 software (SPSS, Inc, Chicago, IL, USA). Raw data are displayed by showing the mean values and standard deviations. Comparisons between the group of study participants and representative norm samples or other comparative samples were carried out using the Welch test due to lack of homoscedasticity. For within-group comparisons, an analysis of variances (ANOVA) was used to describe differences in traumatization (FST, PDS) related to gender, the feeling of integration in the district association, and supervision. Correlations between psychological stress and factors of resilience were calculated using Pearson’s correlation coefficients. A multiple regression analysis was conducted to find variables that predict traumatic stress. Missing values were excluded listwise.

## 3. Results

### 3.1. Secondary and Primary Traumatization

Table 2 depicts the psychological stress of volunteers working in psychosocial emergency care. The volunteers had a mean score of M = 42.68 (SD = 10.52) in the QST/FST which measured their secondary traumatization. The mean score lies below the cut-off score (=65), which means that the participants had no clinically relevant symptoms. Furthermore, the volunteers presented lower levels of secondary traumatization than interpreters working with refugees (M = 52.50, SD = 15.60; t(109) = −4.21, *p* < 0.001; Cohen’s d = −0.74; [47]), trauma therapists (M = 58.00, SD = 18.60; t(172) = −9.26, *p* < 0.001; Cohen’s d = −1.01; [48]), and caregivers for refugees (social workers, psychotherapists, physicians, interpreters; M = 52.94, SD = 16.92; t(141) = −4.57, *p* < 0.001; Cohen’s d = −0.73; [49]). Regarding individual FST scores, only five volunteers (6.7%) suffered from a moderate symptom load (scores between 65 and 82). Female volunteers showed a higher symptoms score of secondary traumatization (M = 44.83, SD = 11.15) than male participants (M = 37.68, SD = 6.66; F(1) = 6.81, *p* = 0.011; Cohen’s d = 0.78). Additionally, volunteers who felt well integrated in their district association (M = 41.87, SD = 10.22) presented less symptoms of secondary traumatization than individuals who did not feel integrated (M = 54.20, SD = 8.35; F(1) = 6.87, *p* = 0.011; Cohen’s d = 1.32). All volunteers were supervised (internally, externally, case discussions, debriefings). For each form of supervision, there were no group differences in secondary traumatization whether the participants had received this form of supervision or not (dichotomous testing; all *p* > 0.05).

Regarding PDS results for primary traumatization, 62 participants (81.3%) stated that they had experienced a traumatic event in the past: 45.3% of the participants had survived an accident, fire, or explosion; 40.0% had suffered from a severe illness or had a relative who had been very sick; and 30.7% had experienced a natural disaster. Of those individuals who had experienced a traumatic event, only one participant (1.3%) showed a low symptom load of primary traumatization (score 10–20), while all other participants did not present any symptoms. Regarding primary traumatization, there was no difference between females (M = 2.83, SD = 3.42) and males (M = 2.28, SD = 2.70; F(1) = 0.42, *p* = 0.520). Also for primary traumatization, the volunteers showed no difference in terms of whether they had received a particular form of supervision or not (all *p* > 0.05).

### 3.2. Psychological Comorbidity

Our results regarding psychological comorbidities are displayed in Table 2. We did not find any significant differences in the level of depressive symptoms measured with the PHQ-9 between the volunteers in psychosocial emergency care (M = 3.31, SD = 2.31) and a German norm sample (M = 3.60, SD = 4.08; t(91) = 1.02, *p* = 0.31; [32]). On an individual level, 73.3% of the participants showed no or minimal depressive symptoms (score 0–4), 25.3% showed mild depressive symptoms (score 5–9), and 1.33% showed moderate depressive symptoms (score 10–14). None of the volunteers suffered from severe depressive symptoms (score 15–27).

The study’s participants presented a lower level of symptoms of anxiety disorders assessed with the GAD-7 (M = 2.49, SD = 1.93) than a German norm sample (M = 2.95, SD = 3.41; t(80) = −2.00, *p* = 0.049; Cohen’s d = −0.17; [33]). Evaluating individual anxiety scores, 88.0% showed no or minimal symptoms of anxiety (score 0–4) and 12.0% showed mild symptoms (score 5–9). None of the participants suffered from moderate (score 10–14) or severe symptoms of anxiety disorders (score 15–21).

Regarding SF-12 scores, participants indicated a better psychological quality of life (MCS, M = 52.16, SD = 8.18) than a German norm sample (M = 50.00, SD = 10.00; t(66) = 2.16, *p* = 0.035; Cohen’s d = 0.24; [50]), whereas the physical quality of life (PCS; M = 48.98, SD = 8.83) did not differ between these two groups (M = 50.00, SD = 10.00; t(66) = 0.70, *p* = 0.487). Of the volunteers, 57.3% reported an average, and 8.0% an above average, psychological quality of life; 73.3% participants revealed an average, and 2.7% an above average, physical quality of life.

### 3.3. Factors of Resilience

The volunteers showed distinct factors of resilience (see Table 3). To the best of our knowledge, this is the first study on psychological stress among volunteers in psychosocial emergency care. Therefore, we compared the volunteers’ resilience factors with norm samples from the general population. The volunteers showed in the ECR-RD questionnaire a significantly lower anxiety of attachment (M = 2.00, SD = 0.76; t(98) = −7.84, *p* < 0.001; Cohen’s d = −0.81) and avoidance of attachment (M = 2.03, SD = 0.81; t(76) = −3.21, *p* <0 0.002; Cohen’s d = −0.36) than a non-clinical sample (M = 2.77, SD = 1.09; M = 2.36, SD = 1.0, respectively; [36]). The assessment via the OPD-SQS questionnaire showed that the volunteers had a higher level of personality functioning (M = 11.52, SD = 6.07) than a non-clinical sample (*N* = 734, M = 15.41, SD = 7.71; t(98) = 5.11, *p* < 0.001; Cohen’s d = 0.56; [39]). The study participants also scored higher in the assessment of sense of coherence via the SOC−29 (M = 155.95, SD = 18.86) than a German norm sample (M = 145.66, SD = 24.33; t(83) = −5.47, *p* < 0.001; Cohen’s d = 0.56; [51]). In the MAAS questionnaire, the volunteers described themselves as more mindful (M = 4.54, SD = 0.70) than a comparative sample of teachers (M = 3.97, SD = 0.70; t(133) = −5.67, *p* < 0.001; Cohen’s d = 0.81). They did not receive more social support (M = 4.69, SD = 0.35; F-SozU) than a representative norm sample (M = 3.97, SD = 0.68; t(0) = −16.83, *p* = 0.999).

### 3.4. Correlations with Secondary or Primary Traumatization

We correlated secondary and primary traumatization with resilience factors and sociodemographic data, and frequency of supervision (external supervision and case discussions). We found a highly significant correlation between secondary traumatization and avoidance of attachment (ECR; *r* = 0.32, *p* = 0.010) and social support (F-SozU; *r* = −0.40, *p* = 0.001). Volunteers who exhibited low levels of avoidance of attachment and/or high levels of social support, also revealed lower results in the assessment of secondary traumatization. Secondary traumatization showed a negative association with case discussions (*r* = −0.29, *p* < 0.05), as well as a positive association with age (*r* = 0.30, *p* < 0.05) and period of volunteering (*r* = 0.25, *p* < 0.05). The more frequently the volunteers discussed cases, the lower the level of secondary trauma was. The older the volunteers were and the longer they had been working in psychosocial emergency care, the higher the level of secondary trauma was. Furthermore, we investigated a highly significant correlation between primary traumatization and mindfulness (*r* = −0.34, *p* = 0.008). Individuals who had high scores in the questionnaire assessing mindfulness displayed lower levels of primary traumatization (see Table 4).

### 3.5. Predictors of Traumatic Stress

Since the correlation of secondary and primary traumatization with some factors of resilience was highly significant, we calculated regression analyses to find out whether those factors of resilience were predictors of traumatization. For secondary traumatization, we also included significant sociodemographic variables as control variables (see Table 5). As potential predictors of secondary traumatization, we included attachment avoidance and social support. As control variables, we added age, gender, period of volunteering, and case discussion. The model explained a variance of 46.9% (R² = 0.469) with significant predictors of gender (B = 5.86, β = 0.26, *p* < 0.05), case discussions (B = −0.52, β = −0.37, *p* < 0.05), and social support (B = −12.34, β = −0.45, *p* < 0.01).

Furthermore, the correlation between primary traumatization and mindfulness (*r* = −0.34, *p* = 0.008) was highly significant. The regression analysis indicated that mindfulness is a significant predictor of primary traumatization (B = −1.59, β = −0.34, *p* = 0.008). The model explained 11.5% of the variance (R² = 0.12, F (1) = 7.53, *p* = 0.008).

## 4. Discussion

We investigated secondary and primary traumatization, comorbid psychological stress, and factors of resilience in volunteers working in psychosocial emergency care. Surprisingly, volunteers in psychosocial emergency care merely showed a marginal symptom load in all the assessed areas. Compared to norm samples, the volunteers displayed no significant differences in depressive symptoms, presented even lower levels of anxiety, had higher levels of psychological quality of life, and reported no differences regarding physical quality of life. Our assessment yielded high scores in the following factors of resilience: Attachment behavior, levels of personality functioning, sense of coherence, and mindfulness. Secondary traumatization correlated highly significantly with avoidance of attachment and social support, as well as frequency of case discussions and period of volunteering. Primary traumatization correlated highly significantly with mindfulness. Regression analysis showed that social support, case discussions, and gender were predictors for secondary traumatization and mindfulness was a predictor for primary traumatization.

As far as we know, this is the first study investigating secondary and primary traumatization in volunteers working in psychosocial emergency care. The results concerning secondary traumatization are in line with existing literature. There are several studies investigating secondary traumatization in other groups of first responders, which have indicated that first responders display very little psychological strain despite regularly being exposed to potentially traumatic situations and providing support to trauma survivors. However, due to the fact that all of these studies applied very heterogeneous methods and different instruments to assess secondary traumatization [3], the results are difficult to compare. In order to classify the results of traumatization in volunteers working in psychosocial emergency care, we compared them to other groups of people with psychological stress that were also assessed with the QST/FST questionnaires. Psychosocial emergency care volunteers showed significantly lower symptoms than interpreters and caregivers working with refugees, as well as trauma therapists and other professionals working with traumatized people. Even though the frequency of emergency responses varies greatly, volunteers in psychosocial emergency care probably have less contact with traumatized people when compared to the other occupational groups working in this field, which could be one explanation of the lower levels of secondary traumatization. Also, the group affiliation to the psychosocial emergency care could have a positive influence on trauma symptoms. Psychosocial emergency care promotes membership of its members, e.g., through a uniform among other things. Group membership in therapies promotes a sense of security, self-esteem, and peer support, normalizes symptoms and difficulties, reduces the feeling of loneliness, and increases the possibility of model learning [52,53,54]. Compared to interpreters, volunteers in psychosocial emergency care have a more active role and are therefore able to influence certain situations, processes, and arrangements. Consequently, they are likely to feel less overwhelmed during their emergency responses. Furthermore, many interpreters come from the same country as the refugees and also have a history of immigration. They are more likely to identify with the refugees or might be confronted with parallels to their own life stories. Compared to interpreters and social workers who are hired for their language and management skills, the volunteers in psychosocial emergency care are specifically selected to work in emotionally challenging situations. Trauma therapists, who might have similar tasks, are well trained for these emotional challenging situations. Furthermore, they are able to influence the therapy sessions and have a closer and more long-term relationship with their traumatized patients. A study of Reinhard and Maercker [17] was able to show low levels of secondary traumatization in medical emergency personnel despite the constant exposition to traumatic situations and work with trauma survivors. This was rated as a kind of immunizing effect. Nonetheless, this interpretation of the results does not wish to imply that one cannot be emotionally resilient in challenging situations and at the same time empathic.

The examined psychosocial emergency care volunteers exhibited no elevated levels of depression compared to a German norm sample. They showed even lower symptoms of anxiety than a German norm sample, and presented an average physical quality of life. Furthermore, their mental quality of life was rated above average. As mentioned above, psychosocial emergency care authorities carefully select potential volunteers regarding their ability to cope with challenging emergency responses. Only if they are perceived to be mentally healthy and emotionally stable enough to work in psychosocial emergency care do volunteers receive comprehensive training.

However, we cannot rule out that the low prevalence of traumatization and comorbid psychological stress in our study sample might not be, at least partially, explained by a positive response bias. This has already been discussed in the existing literature on other groups of first responders [3]. The current professional culture in which first responders work might generally be reluctant to acknowledge symptoms of psychological stress, because this could be interpreted as a weakness of character [55]. Furthermore, individuals might be concerned that they may appear incapable of doing their job [56]. The role of working as a volunteer in psychosocial emergency care could intensify this concern. Additionally, the work of first responders is connected to a certain social status which might prevent first responders from speaking openly about their psychological burden. The personal expectations of challenging situations may even be filtered “through internalized social expectations of toughness and heroisms which could lead to underreporting” [57].

The low symptom load was accompanied by high resilience among the volunteers in the psychosocial emergency care. Furthermore, gender, case discussions, and social support are predictors for secondary traumatization, and mindfulness is a predictor for primary traumatization. These results are in line with existing literature: That women show higher values of secondary traumatization than men have been shown in earlier studies in general [26] and explicitly with first responders [3]. The fact that case discussions have a positive influence on minimizing symptoms of secondary traumatization speaks in favor of the establishment of regular, mandatory meetings. Beyond the exchange of experiences in emergency responses, case discussions promote known resources, such as active problem solving, self-reflection, and skill building [58]. Studies show that factors of resilience, especially social support, prevent first responders from developing secondary traumatic stress [3]. The district associations of psychosocial emergency care organize regular staff meetings, supervision, and follow-up meetings. Furthermore, the volunteers always respond to psychosocial emergency care assignments with a partner. Thus, they are able to support each other during the emergency response and discuss their experiences afterwards. Such events and structures might strengthen this specific group’s cohesion, increase group stability, and therefore, create a feeling of social support. Setti et al. [59] showed that collegial support also has a negative effect on the development of trauma symptoms. The results of the study indicate that volunteers in psychosocial emergency care benefit greatly from social support. Panter-Brick [23] showed that it can be helpful to promote the resources of the organization if one wants to strengthen the resilience of individuals. Therefore, in addition to the individual selection process for volunteers, it might be advisable to ensure that the organizations have sufficient resources at their disposal to support their volunteers and develop their skills.

Limitations: The study participants were all volunteers trained by, and working for, the German Red Cross in the federal state of Baden-Württemberg, Germany. On the one hand, this means that all volunteers were schooled in a comparable way and the sample is homogenous. On the other hand, the results might not be generalizable to other groups. Although we can assume that the training of all volunteers was the same, the type and frequency of the emergency responses, as well as form and frequency of supervision, varied depending on the district association. This restricts the generalizability of the training. The predictors of secondary and primary traumatization were evaluated within a cross-sectional design. In order to confirm the results, longitudinal research would be necessary.

## 5. Conclusions

In this study, the group of volunteers working in psychosocial emergency care presented low levels of secondary and primary traumatization and displayed lower symptoms of anxiety disorders than a norm sample. They showed less psychological strain than people in similar professions, such as interpreters, caregivers working with refugees, or trauma therapists. In addition, the volunteers showed higher psychological quality of life than relevant norm samples. Furthermore, our assessment revealed high factors of resilience which negatively correlated with traumatization: Secondary traumatization was connected to higher levels of avoidance of attachment and lower levels of social support, whereas primary traumatization was connected with lower scores of mindfulness. Female gender, a low frequency of case discussions, and little social support were predictors for secondary traumatization, and mindfulness was a predictor for primary traumatization. In order to confirm these results, longitudinal research would be appropriate.

## Figures and Tables

**Table 1:** Sociodemographic data of volunteers in psychosocial emergency care.

Sociodemographic Data	*n*	%	
Gender			
Male	22	29.3%	
Female	52	69.3%	
Missings	1	1.3%	
Age	M = 51.01	SD = 13.34	
Mother tongue			
German	71	94.6%	
German/Italian	2	2.7%	
English	1	1.3%	
Graduation			
A level	24	32.0%	
Junior high	35	46.7%	
Other	16	21.4%	
Employment			
Full-time	39	52.0%	
Part-time	17	22.7%	
Not	15	20.0%	
Missings	4	5.3%	
Family status			
Married	48	64.0%	
Partnership	11	14.7%	
No partnership	9	12%	
Other	7	9.4%	
Psychosocial emergency care			
Period of volunteering (years)	M = 7.64	SD = 5.54	
No. of emergency responses	M = 62.74	Range: 0–500	
Supervision			
External supervision	Yes: 50 (66.7%)	Frequency: 0.5–6/year	
Case discussions	Yes: 66 (88.0%)	Frequency: 3–60/year	
Supervision by supervisor	Yes: 9 (12%)	Frequency: As required	
Debriefings	Yes: 60 (80.0%)	Frequency: As required	
Adequate amount of supervision	Yes: 66 (90.4%)	No: 7 (9.6%)	
Feeling integrated in the group	Yes: 68 (93.20%)	No: 5 (6.70%)	Missings: 2 (2.7%)

**Table 2:** Traumatization and psychological burden of volunteers in psychosocial emergency care.

	Psychosocial Emergency Care			Norm/Comparative Sample					
Traumatization	*N*	M	SD	M	SD	t	*p*	df	Cohen’s d
FST	68	42.68	10.52	52.50 ^a^	15.60	−4.21	<0.001	109	−0.74
				58.00 ^b^	18.60	−4.21	<0.001	172	−1.01
				52.94 ^c^	16.92	−4.57	<0.001	141	−0.73
PDS	62	2.69	3.22						
	Psychological comorbidity								
PHQ-9	75	3.31	2.32	3.60 ^d^	4.08	1.02	0.312	91	
GAD-7	75	2.49	1.93	2.95 ^e^	3.41	−2.00	0.049	80	−0.17
	Quality of life								
PCS	67	49.23	9.00	50.00 ^f^	10.00	0.70	0.487	66	
MCS	67	52.16	8.18	50.00 ^f^	10.00	2.16	0.035	66	0.24

^a^ Interpreters for refugees, *N* = 64; ^b^ trauma therapists, *N* = 312; ^c^ caregivers for refugees, *N* = 84; ^d^ norm sample, *N* = 2066; ^e^ norm sample, *N* = 5036; ^f^ norm sample, *N* = 21,248.

**Table 3:** Factors of resilience of volunteers in psychosocial emergency care.

	Psychosocial Emergency Care				Norm Sample				
	*N*	M	SD	M	SD	t	*p*	df	Cohen’s d
ECR-RD: Attachment anxiety	70	2.00	0.76	2.77 ^a^	1.09	−7.84	***	98	−0.81
ECR-RD: Attachment avoidance	70	2.03	0.81	2.36 ^a^	1.00	−3.21	**	76	−0.36
RQ-2self	74	5.08	2.93						
RQ-2-other	74	1.95	3.33						
OPD-SQS	74	11.52	6.07	15.41^b^	7.71	−5.11	***	98	−0.56
SOC−29	75	155.95	18.86	143.66 ^c^	24.33	5.47	***	83	0.56
F-SozU	75	4.69	0.35	3.97 ^d^	0.68	16.83		0	
MAAS	71	4.54	0.70	3.97 ^e^	0.70	5.74	***	133	0.81

Note: ^a^ *N* = 1006, ^b^ *N* = 734, ^c^ *N* = 1944, ^d^ *N* = 2507, ^e^ *N* = 157, ** *p* < 0.01; *** *p* < 0.001.

**Table 4:** Pearson’s correlations between traumatization and factors of resilience of volunteers in psychosocial emergency care.

	QST	PDS	ECR-RD: Attachment Anxiety	ECR-RD: Attachment Avoidance	OPD-SQS	SOC−29	F-SozU	MAAS	External Supervision	Case Discussions	Age	Period of Volunteering (years)	No. of Emergency Responses
QST	-	0.33 *	0.20	0.32 **	0.25 *	−0.27 *	−0.40 **	−0.17	−0.22	−0.29 *	0.30 *	0.25 *	0.18
PDS		-	0.27 *	0.12	0.31 *	−0.16	−0.18	−0.34 **	−0.13	−0.16	0.04	0.06	−0.09

* *p* < 0.05; ** *p* < 0.01.

**Table 5:** Regression analyses for predictors of secondary traumatization.

Variable	B	SE B	β	*p*
Constant	90.372	20.237	-	
Age	−0.021	0.098	−0.027	
Gender	5.857	2.555	0.258	*
Period of volunteering (years)	0.147	0.237	0.076	
Case discussions	−0.520	0.162	−0.374	*
ECR—Attachment avoidance	2.784	1.624	0.210	
F-SozU	−12.339	3.559	−0.448	**
R^2^	0.469			
	F(6,47) = 6.928, *p* < 0.001			

* *p* < 0.05; ** *p* < 0.01.
